# Molecular Characterization of Epithelial Ovarian Cancer: Implications for Diagnosis and Treatment

**DOI:** 10.3390/ijms17122113

**Published:** 2016-12-15

**Authors:** Veronica Rojas, Kim M. Hirshfield, Shridar Ganesan, Lorna Rodriguez-Rodriguez

**Affiliations:** 1 Department Obstetrics/Gynecology and Reproductive Sciences, Rutgers Robert Wood Johnson Medical School, 125 Paterson Street, New Brunswick, NJ 08901, USA; vr252@rwjms.rutgers.edu; 2 Department of Medicine, Division of Medical Oncology, Rutgers Robert Wood Johnson Medical School, Rutgers Cancer Institute of New Jersey, 195 Little Albany Street, New Brunswick, NJ 08903, USA; hirshfie@cinj.rutgers.edu (K.M.H.); ganesasn@cinj.rutgers.edu (S.G.); 3 Precision Medicine Oncology, Rutgers Cancer Institute of New Jersey, 195 Little Albany Street, New Brunswick, NJ 08903, USA; 4 Department Obstetrics/Gynecology and Reproductive Sciences, Division of Gynecologic Oncology, Rutgers Robert Wood Johnson Medical School, Rutgers Cancer Institute of New Jersey, 195 Little Albany Street, New Brunswick, NJ 08903, USA

**Keywords:** epithelial ovarian cancer, next-generation sequencing, high throughput sequencing

## Abstract

Epithelial ovarian cancer is a highly heterogeneous disease characterized by multiple histological subtypes. Molecular diversity has been shown to occur within specific histological subtypes of epithelial ovarian cancer, between different tumors of an individual patient, as well as within individual tumors. Recent advances in the molecular characterization of epithelial ovarian cancer tumors have provided the basis for a simplified classification scheme in which these cancers are classified as either type I or type II tumors, and these two categories have implications regarding disease pathogenesis and prognosis. Molecular analyses, primarily based on next-generation sequencing, otherwise known as high-throughput sequencing, are allowing for further refinement of ovarian cancer classification, facilitating the elucidation of the site(s) of precursor lesions of high-grade serous ovarian cancer, and providing insight into the processes of clonal selection and evolution that may be associated with development of chemoresistance. Potential therapeutic targets have been identified from recent molecular profiling studies of these tumors, and the effectiveness and safety of a number of specific targeted therapies have been evaluated or are currently being studied for the treatment of women with this disease.

## 1. Introduction

Ovarian cancer is the leading cause of gynecologic cancer death in developed countries and often presents at an advanced stage [1]. The current standard-of-care for the treatment of the majority of patients with advanced ovarian cancer involves cytoreductive surgery and platinum-based chemotherapy [2]. Despite high response rates for many patients receiving initial chemotherapy, most patients with advanced ovarian cancer ultimately develop recurrent disease that is resistant to chemotherapy. Alternative approaches to the diagnosis and treatment of patients with ovarian cancer are, therefore, urgently needed. This review focuses on recent advances in the molecular characterization of ovarian cancer and the implications for its earlier detection as well as for selection of therapy in patients with refractory or recurrent disease. 

## 2. Ovarian Cancer Screening

Currently, there is no established screening test for ovarian cancer, thereby hindering early stage detection of this disease. An assessment of ovarian cancer screening conducted in 1998 by the National Health Service Health Technology Assessment Programme based on a review of 25 ovarian cancer screening studies was that the routine use of cancer antigen 125 (CA125) serum testing and transvaginal ultrasound was unwarranted [3]. While some evidence suggested that these modalities may be capable of detecting ovarian cancer at an earlier stage in asymptomatic women, the recommendation against routine screening was based on the absence of randomized clinical trials investigating this approach, as well as the lack of evidence regarding its associated benefits, harms and costs. Subsequently, publication of the results of the ovarian arm of the randomized, controlled Prostate Lung Colorectal Ovarian (PLCO) study comparing serum CA125 testing plus transvaginal ultrasound with no screening in over 78,000 women aged 55 to 74 years did not show an ovarian cancer specific survival benefit associated with ovarian cancer screening [4], even with extended follow-up of a median of 15 years [5]. Recently, results from the large UK Collaborative Trial of Ovarian Cancer Screening (UKCTOCS) study have been reported [6]. In this study of over 202,000 postmenopausal women aged 50 to 74 years, subjects were randomly assigned in a 1:1:2 ratio to ovarian cancer screening using a multimodal strategy (MMS) involving serum CA125 testing followed by transvaginal ultrasound as a second-line test in women with serum CA125 values exceeding a pre-established cutoff point, transvaginal ultrasound alone, or no screening [6]. While the primary analysis of the UKCTOCS study data did not reveal a significant reduction in mortality with ovarian cancer screening, a comparison of the MMS and no screening arms showed a trend for decreased mortality in the former group that emerged after year seven. In this context, it is worth noting that insight into the molecular characteristics of ovarian cancer oncogenesis has the potential to enhance early detection of ovarian cancer through multiple approaches, such as the identification of possible precursor lesions, as well as other serum biomarkers (e.g., circulating cell-free DNA or microRNA) in women at high risk for the disease; these topics will be addressed in more detail in subsequent sections.

## 3. Ovarian Cancer as a Heterogeneous Disease

### 3.1. Type I and II Epithelial Ovarian Cancers

**Figure 1:**
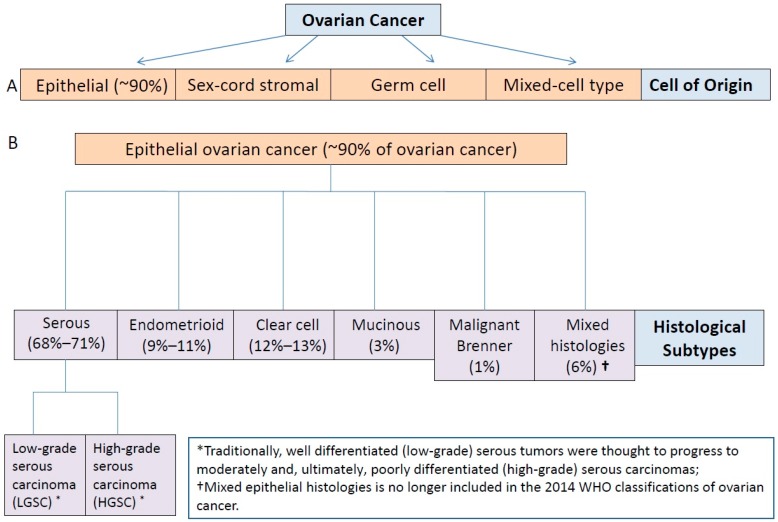
(**A**) Histological subtypes of ovarian cancer; and (**B**) traditional epithelial ovarian cancer classification paradigm based on tumor histology and grade (prevalence of histological subtypes from: McCluggage et al. [8]).

On both histologic and molecular levels, it is becoming increasing clear that ovarian cancer is a clinically diverse and morphologically and molecularly heterogeneous disease [7]. Although the majority of ovarian cancers are classified as epithelial cancer, the histological subtypes of epithelial ovarian cancer include serous, endometrioid, clear cell, mucinous, malignant Brenner tumors, and mixed histologies, with over two-thirds of cases classified as having serous histology (Figure 1) [8,9]. The other histological subtypes of epithelial ovarian cancer are considerably less common compared with high-grade serous carcinoma, although the incidence of ovarian clear cell carcinoma has been reported to be higher in Japan compared with the results of studies conducted on populations in the U.S. and Canada [10,11,12,13].

**Figure 2:**
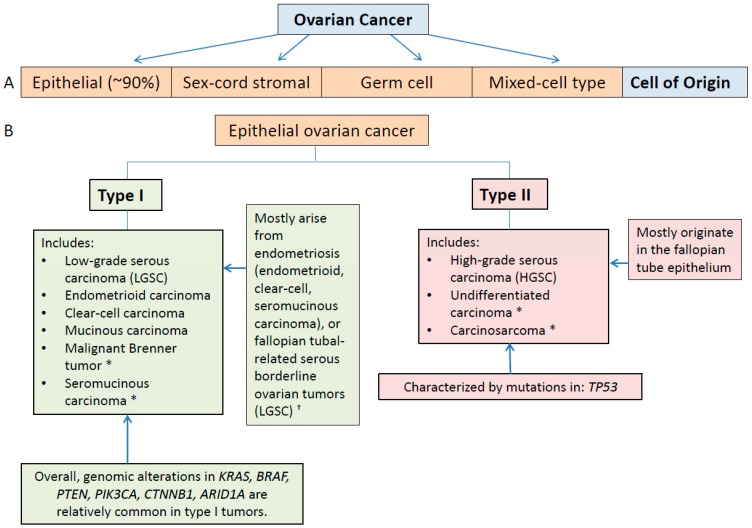
(**A**) Histological subtypes of ovarian cancer; and (**B**) widely accepted epithelial ovarian cancer classification paradigm based on clinicopathologic and molecular evidence that type I and type II tumors develop through different pathways (Kurman et al. [18]). * Indicates rare tumor; ^†^ Mucinous and malignant Brenner tumors are considered to be possible exceptions that may arise from transitional cells at or close to the junction of the fallopian tube and the peritoneum.

Traditionally, high-grade serous carcinoma, a common and typically aggressive form of ovarian cancer, has been considered to arise from a well differentiated, low-grade form of serous carcinoma [14,15]. However, recent advances in tumor molecular characterization correlated with findings from clinicopathologic and molecular characterizations of ovarian cancers have revealed that epithelial ovarian cancer can be defined by two distinct groups termed type I and type II carcinomas (Figure 2) [14,15,16,17,18].

These studies have shown that low-grade serous carcinoma is unlikely to be a precursor lesion for high-grade serous carcinoma; rather, the two conditions are generally believed to be separate diseases characterized by differing patterns of genomic variation, with distinctly different prognostic implications [14,15,18]. Using this newer paradigm, low-grade serous carcinoma is classified as a type I carcinoma. Type I lesions are generally characterized as having a more indolent clinical course and a relatively stable genomic profile [15,18]. High-grade serous carcinoma, on the other hand, is classified as a type II carcinoma and associated with a more aggressive clinical course [15,18]. 

This new classification scheme not only facilitates more accurate characterization of disease and prediction of patient prognosis, it also provides insight into the mechanisms underlying the development of epithelial ovarian cancers. For example, most type I tumors are believed to arise from endometriosis or borderline serous tumors whereas type II tumors are generally believed to principally originate in the fallopian tube [8,14,15,18]. This latter finding is consistent with the advanced stage at which high-grade serous carcinomas are typically detected, and has implications regarding earlier detection of this form of ovarian cancer. 

### 3.2. Molecular Classification and Characterization of Epithelial Ovarian Cancer: Implications for Diagnosis

Molecular studies have uncovered a wide range of genomic variability associated with different histological subtypes of epithelial ovarian cancer, but also have been fundamental to providing the basis for a more simplified approach to organizing these cancers with respect to clinical course and disease origin [15,18]. For example, the more indolent type I tumors, with the possible exception of ovarian clear cell carcinoma, are often characterized by mutations in regulators of the mitogen-activated protein kinase (MAPK) pathway (e.g., *KRAS* or *BRAF*), as well as a number of other genomic variants (Table 1) [15,18,19,20,21,22,23,24,25,26,27,28,29,30,31,32,33,34,35,36,37].

More specifically, in addition to activating mutations in either *KRAS* or *BRAF,* particularly in low-grade serous, endometrioid tumors, and mucinous tumors, alterations of genes encoding β-catenin (e.g., *CTNNB1*), *CDKN2A*, *PIK3CA**,*** and *PTEN* have also been found in a number of studies of type I lesions; mutations in *TP53* are rarely seen in these tumors, with the exception of mucinous carcinoma in which *TP53* mutations occur relatively frequently (Table 1) [15,18,21,24,25,29,33,34,35,36]. Although type I ovarian cancers have been less well characterized by high throughput sequencing studies compared with type II tumors, the findings of these studies have supported the existence of genetic heterogeneity between and within each ovarian cancer histological subtype classified as type I ovarian cancer. For example, a number of studies have revealed a high frequency of inactivating mutations in *ARID1A* (a tumor suppressor gene involved in chromatin remodeling) in ovarian clear cell carcinomas [26,27], and amplification of *ERBB2* (gene encoding for HER2) in 19% and 14% were reported in mucinous tumors and clear cell carcinomas of the ovary, respectively [22,37]. In addition, recent DNA sequencing studies have revealed the presence of somatic and germline mutations in homologous recombination genes in non-serous ovarian cancers, including some type I lesions; these included somatic mutations in *BRCA1*, *BRCA2*, *CHEK2*, *ATM*, and germline mutations in *BRCA1*, *BRCA2*, *RAD51D*, *CHEK2* and *BRIP1*, although *BRCA1/2* alterations were less common compared with serous ovarian cancers [38,39,40]. In one study, a loss of function in at least one homologous recombination gene occurred in 28% of non-serous cases [38]. In contrast, high throughput sequencing studies of the more clinically aggressive type II tumors have generally revealed a high degree of genomic/chromosomal instability, and are characterized by nearly ubiquitous mutations in *TP53* (Table 2) [14,15,16,18]. In fact, a careful review of the few reported cases of *TP53* wild-type high-grade serous ovarian cancer from the TCGA showed that the majority of these tumors were not actually pure high-grade serous ovarian carcinoma [41]. In addition, high rates of somatic and germline genomic defects in *BRCA1/2* and other homologous recombination genes overall, as well as a high percentage of gene copy number variations have been reported in high-grade serous ovarian cancer (Table 2) [16,38,39,40].

Although assignment of epithelial ovarian cancer tumors to either type I or type II categories is currently done on the basis of disease histology and tumor grade, it is reasonable to expect that evolving molecular analyses made possible by more sophisticated DNA and RNA sequencing technologies will continue to provide a more accurate basis for tumor classification in the future, and may further refine the classification of ovarian cancers according to molecular characteristics, leading to more detailed models of the disease. For example, genomic profiling is likely to be very helpful in further classifying ovarian tumors designated as undifferentiated or mixed histology according to histopathological analyses. Furthermore, advanced molecular profiling technologies are likely to serve as the basis for validating the sites of origin of high-grade serous ovarian cancers. More specifically, serous tubal intraepithelial carcinomas (STICs), occurring within the fallopian tubes, are being closely scrutinized as possible precursor lesions of high-grade serous ovarian cancer [7,18,42], and the finding that *TP53* mutations are common in these lesions, and may represent a very early driver mutation, provides support for this proposal [42,43]. Nevertheless, whether STICs represent the sole source of high-grade serous ovarian cancer is still being investigated [44,45,46].

### 3.3. Inter- and Intratumoral Heterogeneity in Ovarian Cancer

As previously detailed, molecular characterization of ovarian cancer specimens has uncovered a wide range of tumor heterogeneity within epithelial ovarian cancer (e.g., inter-patient heterogeneity) that extends to tumors of the same histological subtype. In addition, these types of studies have also demonstrated the existence of both intra- and intertumoral heterogeneity at the level of the individual patient, a phenomenon that has also been observed in other tumor types [47]. Synchronous ovarian cancers that are separated in space (e.g., primary and metastatic lesions) and have distinct molecular profiles are representative of spatial intertumoral heterogeneity. Intratumoral heterogeneity is another example of spatial heterogeneity and is likely to occur through subclonal tumor evolution [48,49,50]. Temporal heterogeneity in the molecular profiles of ovarian cancer tumors can also occur. For example, changes in the molecular profiles of ovarian cancer tumors may occur over time (e.g., at diagnosis vs. relapse), probably in response to the selection stress of specific therapeutic regimens [50,51]. 

Next-generation/high-throughput sequencing has been used to investigate tumor evolution of specimens of high-grade serous ovarian cancer [40,48,49,51,52,53], as well as non-serous ovarian cancer lesions [40]. These studies have allowed for the construction of phylogenetic trees for the assessment of evolutionary patterns representing the intrinsic diversity of subclonal populations within treatment-naive ovarian cancer tumors and, in some cases, tumor evolutionary response to treatment. Some of the major findings from these studies include the demonstration of significant genomic variation between different regions of the same tumor and/or between synchronous primary and metastatic tumors, even in untreated patients [40,48,49,52,53]. Furthermore, a high degree of spatial heterogeneity was also observed in type I ovarian cancer tumors [39,40]. Regarding temporal heterogeneity, some studies of high-grade ovarian serous cancer show that most clonal characteristics evident in recurrent or metastatic disease are present as subclonal populations within the primary tumor [40,49,52], although one study showed evidence of ongoing tumor evolution [51]. In this context, it is worth noting that assays for sequencing tumor cell-free circulating DNA from the bloodstream, while still evolving, have the potential to detect ovarian cancer at an early stage, to provide simultaneous genomic information on multiple tumor foci and to reveal ongoing changes in tumor genomic characteristics that occur following surgery and subsequent therapy, thereby circumventing many of the limitations imposed by direct sampling of heterogeneous tumors [54]. In addition, next-generation/high throughput sequencing studies of STIC specimens may provide an opportunity to uncover molecular processes involved in tumorigenesis and the development of tumor heterogeneity in the setting of high-grade serous ovarian cancer [7]. 

Beyond DNA sequencing, the molecular characterization of epithelial ovarian cancers at the level of mRNA expression has the potential to identify and quantify specific transcripts expressed within the tumor specimen at a given point in time, and to provide an enhanced understanding of tumor evolution on a detailed molecular level. Gene expression profiling studies conducted within the last decade have focused on the elucidation of differential gene expression between epithelial ovarian cancer and normal ovarian tissue using RNA microarray analysis. These studies have provided evidence for the existence of several distinct molecular subtypes of high-grade ovarian cancer (i.e., immunoreactive, differentiated, proliferative, and mesenchymal) that correlate with clinical outcome [55,56,57]. A similar type of assay was used to determine whether more than one molecular subtype could be detected when tumor located in different anatomic locations (i.e., right and left ovary, omentum, and peritoneal metastases to the bladder or uterus) in individual chemo-naive patients were compared [58]. While most of the tumor subtypes were unchanged across anatomic locations, primary tumors classified as proliferative subtype showed substantial heterogeneity across multiple anatomic sites. However, results from another similar study revealed that individual high-grade serous ovarian tumor specimens often expressed multiple subtype signatures [59]. One limitation of these types of studies is that they provide only an indirect determination of the sequence of complementary DNAs (cDNAs) and cannot detect novel genes and isoforms. More recently, a systematic analysis of the transcriptomes of a large number of specimens of high-grade serous ovarian cancers acquired using high throughput RNA sequencing (RNA-seq) was reported [60]. This study was designed to uncover specific mRNA isoforms that can be classified as being highly or exclusively expressed in ovarian cancer, but not in normal tissue, and the results revealed that the tumors were frequently characterized by expression of *ETV4*, *FOXM1*, *LSR*, *CD9*, *RAB11FIP4*, and *FGFRL1*. However, the authors stated that further work is needed before therapeutic applications of these findings are elucidated.

### 3.4. Molecular Classification of Chemoresistant Epithelial Ovarian Cancer

Genomic heterogeneity can occur as the result of selective pressures (e.g., chemotherapy) that induce changes to the molecular landscape of epithelial ovarian tumors. A study involving whole genome sequencing of specimens from patients with chemoresistant high-grade serous ovarian cancer has recently been reported [61]. An advantage of whole genome sequencing over an approach focused on sequencing exons only is that it detects structural aberrations that may occur outside coding areas of the genome, such as translocations and rearrangements, including gene breakages and gene fusions; it also provides a more direct and accurate assessment of copy number alterations as well as of the exome itself. This study is particularly notable for two reasons: it is the first study to completely characterize the genome of a common form of ovarian cancer that is associated with a poor prognosis and very limited treatment options, and it provides more comprehensive insight into the genomic heterogeneity of high-grade serous ovarian cancer that has evolved under the selective influence of a specific type of therapeutic approach. The results of this study showed that acquired resistance to chemotherapy is associated with inactivating gene breakages in tumor suppressors *RB1*, *NF1*, *RAD51B*, and *PTEN*, and that resistant/refractory disease was frequently associated with a relatively high frequency of *CCNE1* amplification. Moreover, the low frequency of point mutations observed in earlier next-generation/high throughput sequencing studies of high-grade serous ovarian cancer was also observed in chemoresistant recurrent disease. In this study, which also involved matched sequencing of germline DNA samples, there were cases of reversions in germline *BRCA1* or *BRCA2* mutations as well as loss of *BRCA1* promoter hypermethylation in the setting of acquired resistance. In addition, the *ABCB1* gene encoding for the multi-drug-resistant protein 1 (MDR1) was upregulated as a result of promoter translocation and fusion events in approximately 8% of samples of patients with recurrent disease. The results of this study, as well as several other recent studies utilizing next-generation/high throughput sequencing technology to characterize molecular changes in high-grade serous ovarian tumors over time in response to the selective pressure of chemotherapy suggest that both selection of subclones present in the primary tumor as well as acquisition of a limited number of new genomic alterations may be associated with the development of acquired chemoresistance in patients with recurrent high-grade serous ovarian cancer [40,49,51,61,62]. Interestingly, a very recent whole exome sequencing study of 31 high-grade serous ovarian cancer specimens did not show evidence of reactivating mutations in *BRCA1* or *BRCA2* in platinum-sensitive, relapsed tumors after a single line of standard-of-care, platinum-based chemotherapy compared with untreated primary tumors [62]. 

## 4. Implications of the Molecular Characterization of Epithelial Ovarian Cancer for Selection of Targeted Therapy

One of the main objectives of developing a molecular classification of epithelial ovarian cancer that reflects the underlying pathogenesis of the disease is to facilitate individualized treatment selection. However, despite the complexity of ovarian cancer, the current standard-of-care for the treatment of this disease is only minimally dependent on histological subtype or other disease classification scheme, and platinum-based chemotherapy is currently recommended as primary systemic therapy for most patients with epithelial ovarian cancer [2]. Furthermore, these treatment recommendations, particularly with respect to type I ovarian cancers, are often based on limited evidence of efficacy. Use of the dichotomous classification paradigm (i.e., types I and II), based in part on the genomic characterization of ovarian cancers, has provided a possible pathophysiologic explanation for the primary chemoresistance often associated with type I lesions which are considered to be more genomically/chromosomally stable than type II ovarian cancer tumors [15,16,17,18]. Nevertheless, even high-grade serous ovarian cancer, categorized as a genomically unstable, type II lesion [15,16,17,18], frequently recur following an initial response to chemotherapy, and, as discussed above, some of the molecular changes associated with this type of chemoresistance have recently been revealed using next-generation/high throughput sequencing technology [49,51,61,62]. Furthermore, there is no standard-of-care for the treatment of epithelial ovarian cancer that is either refractory or acquires resistance to platinum-based chemotherapy. 

Although molecularly-targeted therapies are urgently needed for patients with ovarian cancer, these types of treatment approaches necessitate knowledge of one or more driver mutations that alter the function of a limited number of cellular signaling pathways. In addition, selection of targeted therapy is based on the premise that these genomic alterations are “actionable” in the sense that they have significant therapeutic implications in subsets of patients with ovarian cancer and for specific therapies. At the present time it is likely that only a limited portion of the highly complex molecular information available through the detailed molecular characterization of epithelial ovarian cancers by next-generation/high throughput sequencing will be translatable into targeted therapeutic approaches [7]. Moreover, it is possible that the existence of multiclonal disease and tumor subclonal evolution may serve to decrease the effectiveness of a particular targeted therapy if at least some of the subclones are representative of true tumor heterogeneity and have additional genomic alterations that drive tumorigenesis [63]. In addition, selection pressure exerted by treatment may alter the relative proportions of subclonal populations, ultimately leading to treatment resistance [64]. Hence, a therapeutic approach involving successively altered combinations of targeted agents that are selected in response to dynamic molecular changes occurring in the tumor may be a more logical strategy in the treatment of recurrent or chemoresistant ovarian cancer. Such a strategy is based on molecular findings observed across serial biomarker assessments performed at tumor progression. Another potential treatment approach may involve therapy directed against targets demonstrated, through longitudinal sequencing studies of ovarian cancer tumors, to be less affected by clonal evolution and to occur more consistently within those tumors.

Presently, only two targeted agents have received approval for the treatment of ovarian cancer by the U.S. Food and Drug Administration and the European Medicines Agency (EMA). These are the polyadenosine diphosphate (ADP)-ribose polymerase (PARP) inhibitor, olaparib, and the antiangiogenic agent, bevacizumab, both of which are approved for patients with advanced disease that has been pretreated with chemotherapy [65,66]; first-line bevacizumab for the treatment of ovarian cancer has also been approved by EMA [67]. In addition trabectedin, an inhibitor of DNA replication and transcription and an inducer of DNA double-strand breaks and loss of homologous recombination repair [68], is also available in Europe for the treatment of patients with advanced ovarian cancer [67].

### 4.1. Molecular Targets for Therapy in Ovarian Cancer

**Figure 3:**
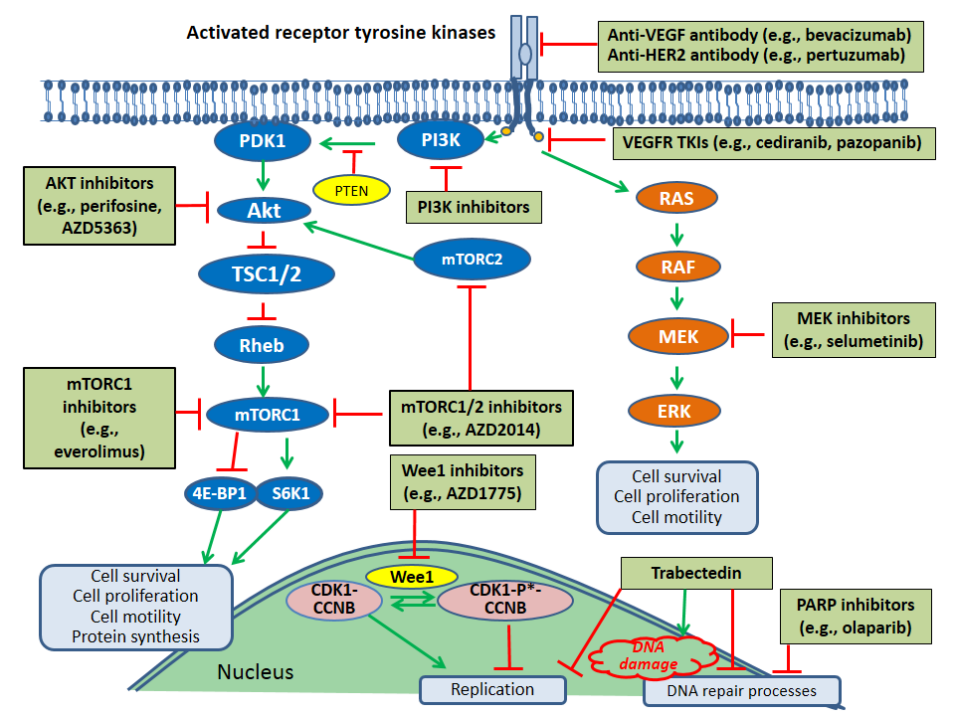
Molecular targets in ovarian cancer treatment; * Indicates inactive (phosphorylated) form of cyclin-dependent kinase 1; Green arrows indicate stimulation while the red lines indicate inhibition; 4E-BP1: eukaryotic translation initiation factor 4E-binding protein 1; AKT: protein kinase B; CCNB: cyclin B; CDK1: cyclin dependent kinase-1; ERK: extracellular signal-related kinase; HER2: human epidermal growth factor receptor 2; MEK: MAPK/ERK kinase; mTORC1: mammalian target of rapamycin complex 1; mTORC2: mammalian target of rapamycin complex 2; PDK1: phosphoinositide-dependent kinase-1; PI3K: phosphoinositol 3-kinase; RAF: a serine/threonine-specific kinase; RAS: a member of a specific GTPase superfamily; Rheb: Ras homolog enriched in brain protein; S6K1: S6 kinase beta-1; TSC1/2: tuberous sclerosis proteins 1 and 2; VEGF: vascular endothelial growth factor; VEGFR: vascular endothelial growth factor receptor.

Multiple potential therapies targeted against specific molecular alterations are currently being investigated for the treatment of ovarian cancer, although it is likely that they will be useful in particular patient subsets only. The molecular targets and associated targeted therapies listed in the section below illustrate the diversity of therapeutic approaches that are being evaluated in advanced ovarian cancer, although this list is not meant to be an all-inclusive. Figure 3 depicts molecular pathway components that are targeted by many of these therapies. Of note, crosstalk between the RAS/RAF/MEK/ERK pathway and the mTOR pathway can also occur (i.e., ERK suppression of the tumor suppressor, TSC1/2, indirectly stimulates mTOR complex 1) [69]. 

#### 4.1.1. *BRCA1/2* Mutations

PARP inhibition has been shown to lead to an accumulation of DNA strand breaks, and is believed to be particularly effective in patients with ovarian cancer who have mutations in the *BRCA1* or *BRCA2* tumor suppressor genes since those molecular alterations are also associated with impairment of homologous recombination in DNA repair. More specifically, use of PARP inhibitors in patients with tumors characterized by deficient homologous recombination is an example of synthetic lethality, cell death resulting from a combination of mutations in two or more genes. In addition, there is evidence that the combination of a PARP inhibitor with a platinum agent, which also damages DNA and interferes with its repair, may provide another level of synergism [70,71].

Regulatory approval of olaparib for the treatment of advanced ovarian cancer is based on results of a nonrandomized phase II trial of 298 patients with solid tumors and germline *BRCA1* or *BRCA2* mutations, including 193 patients with recurrent ovarian cancer [72,73]. In the subgroup of patients with ovarian cancer, the majority of whom were heavily-pretreated with chemotherapy, median progression-free survival (PFS) was seven months and median overall survival (OS) was 16.6 months [73]. Other PARP inhibitors in development for the treatment of patients with ovarian cancer include veliparib, niraparib, and rucaparib [74,75]. More recently, the phase III ENGOT-OV16/NOVA study investigated niraparib maintenance therapy or placebo in patients with platinum-sensitive recurrent ovarian cancer previously treated with two or more platinum-based regimens who were stratified according to whether they did or did not have a germline mutation in *BRCA1/2* [76]. Although the subgroup of patients with germline *BRCA1/2* mutations experienced a substantial PFS benefit with niraparib therapy compared with placebo (21.0 vs. 5.5 months; hazard ratio = 0.27; 95% confidence interval (CI), 0.17 to 0.41; *p* < 0.001), perhaps even more notable was the finding that in the niraparib arm, the cohort of patients without a germline *BRCA1/2* mutation also experienced a marked benefit vs. placebo (median PFS 9.3 vs. 3.9 months; hazard ratio = 0.38; 95% CI, 0.34 to 0.61; *p* < 0.001). In this latter group, niraparib-associated PFS benefit was more pronounced in patients with somatic mutations in *BRCA1/2* or other defects in homologous recombination, emphasizing the potential utility of biomarkers other than *BRCA1/2* in selecting patients for treatment with PARP inhibitors. However, long-term benefit of niraparib was also observed in approximately 20% of the patients without either of these biomarkers. Overall, niraparib was well tolerated although grade 3/4 hematologic toxicities were observed in approximately 20% to 33% of patients receiving niraparib. 

In this context, it is also worth noting the data from a recently published exploratory analysis of patients enrolled in the phase III OVA-301 study investigating the use of pegylated liposomal doxorubicin with or without trabectedin in patients with recurrent ovarian cancer. In this analysis, the response rate, PFS and OS for the subgroup of patients with germline *BRCA1* mutations in the trabectedin arm were significantly higher compared with this subgroup of patients receiving chemotherapy alone. However, in the subgroup of patients classified as *BRCA1* wild-type, the addition of trabectedin to chemotherapy was not associated with a difference in OS compared with chemotherapy alone [77]. A phase III clinical trial is now underway to prospectively evaluate this hypothesis [78].

#### 4.1.2. VEGF Pathway 

The molecular rationale for the use of antiangiogenic therapy is based on the results of studies showing that increased levels of vascular endothelial growth factor (VEGF) in ovarian cancer were associated with increased resistance to chemotherapy and a poorer prognosis [79]. Bevacizumab, an anti-VEGF-A antibody, in combination with chemotherapy has been shown to increase PFS compared with chemotherapy alone in patients with recurrent disease, although no OS difference was observed between the two treatment arms [80,81,82]. Overall, bevacizumab has been shown to be reasonably well-tolerated in heavily-pretreated patients with recurrent ovarian cancer [83]. In addition, the combination of bevacizumab with upfront platinum-based chemotherapy followed by bevacizumab maintenance therapy has also been associated with a PFS, but not an OS, benefit in the setting of advanced ovarian cancer [84,85,86]. The use of bevacizumab in combination with platinum-based chemotherapy in the first-line setting or in combination with chemotherapy in pretreated patients with recurrent disease is considered to be a possible treatment option, although not an essential component of standard-of-care treatment [2]. In this context, it is worth noting the results of a recent case-control study of 222 women with advanced ovarian cancer in which women treated with first-line carboplatin/paclitaxel plus bevacizumab were matched with women treated with first-line carboplatin/paclitaxel without bevacizumab [87]. While a seven-month PFS advantage was observed in the patients treated with bevacizumab, this group also experienced more multifocal disease at relapse, as well as a lower rate of secondary cytoreductive surgery, and a lower response rate and a shorter time to progression for second-line chemotherapy. Future analyses of data from the randomized clinical trials involving bevacizumab use in the first-line setting would be better able to address potential confounding factors and should provide more insight into these observations. There is retrospective evidence from the Gynecologic Oncology Group (GOG) study 0218 trial that a phenotypic marker (i.e., ascites) is associated with significant PFS and OS benefit in women with advanced ovarian cancer receiving bevacizumab-containing, first-line chemotherapy followed by maintenance therapy with bevacizumab [88].

A current drawback with the use of antiangiogenic therapy is the lack of a molecular biomarker to identify patients most likely to benefit from such an approach. However, recent studies investigating response to bevacizumab in molecularly-defined subgroups of women with ovarian cancer have provided initial support for the feasibility of such a molecularly-guided approach [89]. For example, an evaluation of the gene expression profiles of patients with high-grade serous ovarian cancer enrolled on the phase III ICON7 trial of woman with advanced ovarian cancer receiving first-line chemotherapy with or without bevacizumab showed that the subgroup of patients with tumors characterized by angiogenic gene repression and immune gene upregulation who received bevacizumab had worse PFS and OS compared with those receiving chemotherapy alone, whereas a trend toward improved PFS with the addition of bevacizumab was seen in the subgroup of patients with tumors characterized by high expression of angiogenesis-related genes [90].

Tyrosine kinase inhibitors that specifically target the VEGF receptor or act as multi-targeted receptor tyrosine kinase inhibitors, including cediranib, pazopanib, and sunitinib, are also under investigation in the setting of advanced ovarian cancer [91]. For example, in a recently reported phase III trial, patients with relapsed platinum-sensitive ovarian cancer were randomly assigned to one of three treatment arms: platinum-based chemotherapy plus placebo followed by placebo only maintenance therapy (arm A); cediranib plus platinum-based chemotherapy followed by placebo only maintenance therapy (arm B); and cediranib plus platinum-based chemotherapy followed by cediranib maintenance therapy (arm C) [92]. Median PFS in the three treatment arms was 8.7 months, 9.9 months, and 11.0 months, respectively, with a hazard ratio of 0.56 (95% CI, 0.44–0.72, *p* < 0.0001) for the comparison of arms C and A, although toxicity was increased for patients receiving cediranib. Interestingly, results from a recent phase II study have demonstrated that PFS in patients with recurrent, platinum sensitive high-grade serous ovarian cancer was significantly longer (17.7 months vs. 9.0 months; hazard ratio = 0.42; 0.23–0.76; *p* = 0.005) in patients receiving the combination of cediranib and olaparib compared with olaparib alone, suggesting that the two agents may act synergistically [93].

#### 4.1.3. PI3K/Akt/mTOR Pathway

Alterations associated with genes encoding for components of the PI3K/Akt/mTOR signaling network (e.g., *PIK3CA* and *PTEN*), a prototypic survival pathway, have been identified in type I and type II tumors (Table 1 and Table 2) [16,20,23,24,25,34,35,36]. Therapies directed against specific components within this pathway have the potential to interrupt its constitutive signaling [17,94,95]. For example, single-agent mTOR inhibition therapy, such as temsirolimus, has shown modest activity in epithelial ovarian cancer [96,97,98]. However, genomic markers predictive of response to mTOR pathway inhibitors across cancers are only beginning to emerge and have not been established [99,100]. Furthermore, inhibition of the mTOR pathway may be more effective when an mTOR inhibitor is administered in combination with chemotherapy or another targeted agent, given the complexity of the mTOR pathway which allows for its activation through multiple pathway components. A number of combination therapy approaches involving this class of agents, including the combinations of everolimus and letrozole and everolimus and bevacizumab, are under investigation in ovarian cancer [101,102,103]. In addition, PI3K and Akt inhibitors, acting upstream of mTOR inhibitors, are in clinical development for the treatment of ovarian cancer and may have the potential to more effectively disrupt this pathway [104,105]. For example, results of a phase I trial of perifosine, an Akt inhibitor, administered in combination with docetaxel showed evidence of clinical activity with a good safety profile in patients with platinum- and taxane-resistant or refractory epithelial ovarian cancer [106]. In addition, a recent phase I study evaluating the combination of an Akt inhibitor, AZD5363, plus the PARP inhibitor, olaparib, as a potential strategy for overcoming resistance to PARP inhibition showed responses in a patient with *BRCA1*-mutant ovarian cancer and also in another with *BRCA1/2* wild-type ovarian cancer [105]. Beyond demonstrating the potential usefulness of an approach that simultaneously targets these two molecular processes, the early results of this study suggest that a *BRCA* mutation may not be the only a determinant of response for patients receiving this combination, and underscore the importance of evaluating new targeted treatment approaches within the context of a clinical trial. Finally, an early-phase clinical trial evaluating a dual mTOR1/2 inhibitor in combination with olaparib in gynecologic cancer is also underway [107]. 

#### 4.1.4. *TP53* Mutations

The tumor suppressor p53 is involved in the regulation of response to stressors including oxygen deficiency, activation of oncogenes, and DNA damage through control of a number of signaling pathways involved in programmed cell death, cell cycle arrest, and cell aging [108]. The very high rate of *TP53* mutations observed in high-grade serous carcinoma make this genomic alteration a very attractive potential therapeutic target (Table 1) [16]. 

A recent phase I study of AZD1775, an inhibitor of the Wee1 G2 checkpoint kinase, administered as a single agent, demonstrated partial responses in patients with refractory solid tumors, including a patient with *BRCA1/2*-mutated ovarian cancer [109]. Two randomized phase II clinical trials investigating AZD1775 in combination with chemotherapy in patients with *TP53*-mutated epithelial ovarian cancer have shown some promising anti-tumor activity for the combination [110,111]. The rationale for this approach is based on the roles played by p53 and Wee1 in the G1 and G2 DNA damage checkpoints, respectively [112]. Tumor cells with impairment in the G1 checkpoint may be more vulnerable to AZD1775, especially when it is administered concomitantly with chemotherapy [113]. In addition, a clinical trial of kevetrin, an activator of p53, has been conducted in patients with advanced solid tumors [114]. 

Another novel method of targeting *TP53* mutations involves inhibition of heat shock protein 90 (Hsp 90) which has been shown to form a stable complex with mutant p53, preventing the degradation of the latter protein, thereby resulting in its accumulation [115,116]. A two-part, multicenter, phase 1/2 clinical trial evaluating the Hsp 90 inhibitor, ganetespib, as a single agent and in combination with paclitaxel in patients with high-grade serous, high-grade endometrioid, or undifferentiated, platinum-resistant epithelial ovarian, fallopian tube or primary peritoneal cancer is underway [117].

#### 4.1.5. RAS/RAF/MEK/ERK Pathway

Although approaches to targeting components of the RAS/RAF/MEK/ERK pathway in epithelial ovarian cancer have not been explored to a great extent, they may be particularly relevant to the treatment of type I lesions given the relatively high frequency of *KRAS* and *BRAF* mutations found in many of these tumors (Table 1). Results from an open-label phase II study of the MEK inhibitor, selumetinib, in 52 patients with recurrent low-grade ovarian serous carcinoma showed an either an objective response (15.4%) or stable disease (65%) in over 80% of patients [118]. In addition, selumetinib was well tolerated in this patient population. Nevertheless, results of an exploratory analysis revealed that the *BRAF* and *KRAS* mutational status of the tumors did not appear to correlate with response to selumetinib [118]. A recent planned interim analysis of data from the phase III MILO study of the MEK inhibitor, binimetinib, in patients with low-grade serous ovarian cancer did not show a significant difference in PFS compared with chemotherapy, although a tumor mutation in a component of the RAS/RAF/MEK/ERK pathway was not an inclusion criterion for study enrollment [119,120]. A randomized phase II/III study of another MEK inhibitor, trametinib, compared with standard therapy in women with recurrent or progressive low-grade serous carcinoma is underway, with plans to characterize tumor mutational status and correlate it to treatment response [121]. 

#### 4.1.6. Other Receptor Tyrosine Kinase Pathways: HER2

An understanding of the significance of HER2 expression in epithelial ovarian cancer is evolving [122]. Rates of objective response in clinical trials of single agent trastuzumab, pertuzumab or lapatinib in patients with advanced, refractory ovarian cancer have been modest, and no significant change in median PFS was observed with the addition of pertuzumab to carboplatin/paclitaxel in a randomized phase II study of patients with relapsed, platinum-sensitive ovarian cancer [123,124,125,126]. However, data from a phase II trial suggest that the combination of pertuzumab with gemcitabine is active in patients with platinum-resistant disease [127]. In this context, it is worth noting the relatively low frequency of *ERBB2* amplification or mutation observed in high-grade serous ovarian cancer [16] (Table 2). Furthermore, the higher frequencies of amplification in *ERBB2* reported in some studies of clear cell and mucinous carcinomas of the ovary suggest that anti-HER2 directed therapy may be more beneficial in these subgroups (Table 1 and Table 2) [22,37,122]. 

#### 4.1.7. MicroRNAs

MicroRNAs (miRNAs) are short, non-coding RNAs that are thought to function as posttranscriptional gene regulators that can either suppress or stimulate tumor growth [128]. Differential expression of miRNAs observed in some cancers, including ovarian cancer, compared with normal tissue has led to the proposal that these molecules may contribute to tumorigenesis [128]. In one study, RNA microarray expression data from the TCGA were used to investigate correlations between expression of specific miRNAs and mRNAs in ovarian cancer in order to identify potential mRNA targets of miRNAs [129]. Specific miRNA-based signatures or networks have been identified in epithelial ovarian cancers characterized by a poor prognosis, and have the potential to serve as prognostic markers in both early and advanced stages of the disease [130,131,132]. In addition, stable circulating cell-free miRNAs that closely mimic the miRNA profiles of tumor have been isolated from the serum and plasma of women with ovarian cancer [133]. Furthermore, distinctly different circulating miRNA signatures were observed in women with ovarian cancer compared with healthy controls, suggesting that circulating miRNAs may be useful as markers for early detection, as well as for disease prognosis [133,134]. Finally, therapeutic strategies involving either replacement or suppression of specific miRNAs have been proposed, although there are currently no clinical trials investigating miRNAs in ovarian cancer. 

#### 4.1.8. Immunologic Pathways

The concept of targeting the immune system to attack cancer cells has recently emerged as a promising new avenue for the treatment of ovarian cancer. Immune cells can recognize and destroy aberrantly proliferating cells, and this activity is modulated by immune checkpoints expressed on T-cells [135,136]. Increased expression of inhibitory immune checkpoints in the tumor’s microenvironment can restrain immunologic responses, enabling cancer cells to evade attack by the immune system, thereby facilitating unrestrained cell growth [135,136]. 

Data from a phase II trial examining the safety and efficacy of nivolumab, an antibody against the programmed cell death-1 (PD-1) immune checkpoint, in 20 patients with platinum-resistant ovarian cancer were recently published [137]. Treatment-related grade three and four and serious adverse events occurred in 40% and 10% of patients, respectively. Fifteen percent of patients experienced an objective response, the disease control rate was 45%, and median OS was 20.0 months. Tumor specimens from the majority (80%) of patients enrolled in the study exhibited high expression of the programmed cell death receptor-ligand 1 (PD-L1) which, when bound to PD-1, interferes with T cell activation. However, 87.5% of the 16 patients who exhibited high programmed cell death-ligand 1 (PD-L1) expression levels, did not respond to nivolumab, indicating that high PD-L1 expression may not be a sufficiently sensitive predictor of response to PD-1 inhibitors. Studies have suggested that the presence of tumor infiltrating lymphocytes, high mutational burden within the tumor genome, or mismatch repair defects may be additional factors to aid in the prediction of response to inhibitors of immune checkpoints [138]. Other immune checkpoint inhibitors, including antibodies against cytotoxic tumor lymphocyte antigen 4 (CTLA4), are also being evaluated for the treatment of patients with ovarian cancer [135,139]. 

Alternative immune-based approaches in development for the treatment of ovarian cancer include vaccines, cytokines, and adoptive T-cell therapy, a treatment involving lymphocyte transfusion [140]. As more information about the subset of genes involved in the molecular regulation of immune function become available, it is likely that next-generation/high throughput sequencing will play an increasing role in the selection of patients with ovarian cancer for therapy targeted to the immune system.

## 5. Conclusions

The high degree of heterogeneity associated with epithelial ovarian cancer has long been an impediment to its effective characterization and to optimizing the treatment of patients with this disease. However, molecular characterization of different histological subtypes of epithelial ovarian cancer, including results of more recent studies utilizing next-generation/high throughput sequencing technology, have facilitated the development of a more unified approach to the classification of these tumors. In conjunction with multiple tumor sampling separated through space and time, this technology is likely to be fundamental in uncovering evolutionary patterns involved in the etiology of tumorigenesis in epithelial ovarian cancer, particularly the molecular processes underlying the pathogenesis of high-grade serous ovarian cancer, as well as the site(s) of precursor lesions and the basis for the development of metastatic disease. 

The limited effectiveness of most single-agent targeted therapies evaluated thus far and the absence of a standard-of-care for patients with advanced epithelial ovarian cancer that is resistant/refractory to platinum-based chemotherapy represent major unmet needs. While the recent strides made in uncovering molecular details of epithelial ovarian cancer have been considerable, the clinical implications of this information are only beginning to be understood. For example, new avenues being opened up through the use of next-generation/high throughput sequencing technology include approaches to characterize the genes responsible for drug resistance in order to facilitate personalized selection and targeted delivery of treatment to suppress the development of such resistance [61,141]. It is, however, clear that overcoming disease resistance to treatment will require a diversity of approaches. Furthermore, it is critically important that the putative drivers of individual tumors uncovered in molecular studies are linked with the discovery of new single-agent and combination targeted regimens in the setting of clinical trials, such as those targeting multiple therapeutic pathways based on the specific molecular characteristics of the disease in individual patients [142,143]. 

## Figures and Tables

**Table 1:** Type I ovarian cancers: Frequencies of selected potentially pathogenic genomic alterations.

Gene Alterations	Low-Grade Serous Cancer	Ovarian Clear Cell Carcinoma	Endometrioid	Mucinous
Mutations				
*BRAF*	33% ^a^; 38% ^b^; 16% ^c^	0% ^e^; 1% ^f^	24% ^a^	0% ^k^; 23% ^l^; 5% ^m^;
*KRAS*	19% ^b^; 35% ^a^; 21% ^c^	<1% ^a^; 7% ^f^	<1% ^a^	50% ^k^; 68% ^n^; 65% ^m^
*PIK3CA*	11% ^b^	25% ^e^; 33% ^f^	12% ^e^	14% ^m^
*PTEN*	20% ^d^	0% ^e^; 5% ^f^	14% ^j^; 31% ^e^	3% ^m^
*ARID1A*	--	46% ^g^; 57% ^h^	30% ^g^	9% ^l^
*CTNNB1*	--	0% ^e^; 3% ^f^	23% ^e^; 24% ^j^	5% ^m^
*CDKN2A*	--	--	--	19% ^m^
*TP53*	--	--	--	57% ^m^; 52% ^l^
Copy number alterations				
*ERBB2* (HER2; gain)	--	14% ^i^	--	12% ^m^; 19% ^o^

^a^ Singer et al. [29]; ^b^ Jones et al. [20]; ^c^ Hunter et al. [32]; ^d^ Landen, et al. [23]; ^e^ Willner et al. [25]; ^f^ Kuo et al. [24]; ^g^ Wiegand et al. [27]; ^h^ Jones et al. [26]; ^i^ Tan et al. [22]; ^j^ Catasus et al. [34]; ^k^ Gemignani et al. [30]; ^l^ Ryland et al. [35]; ^m^ Mackenzie et al. [36]; ^n^ Cuatrecasa et al. [31]; and ^o^ Angelesio et al. [37]; HER2: human epidermal growth factor receptor 2; -- Dashed lines indicate that data are unavailable or not included.

**Table 2:** Selected genomic alterations and their frequencies in high-grade serous ovarian carcinoma from the TCGA ^a^.

Gene	Frequency of Mutations	Frequency of Copy Number Alterations ^b^
*TP53*	96%	0.9%
*BRCA1* ^c^	12%	0.6%
*BRCA2*	11%	2%
*MYC*	0%	31%
*MECOM*	0.6%	22%
*CCNE1*	0%	20%
*PRKCI*	0.6%	19%
*EIF5A2*	0%	18%
*PIK3CA*	0.6%	17%
*NOTCH3*	0.9%	11%
*KRAS*	0.6%	11%
*RAB25*	0%	7%
*AKT2*	0%	6%
*AURKA*	0%	3%
*PIK3R1*	0.3%	2% ^d^
*AKT1*	0%	3%
*ERBB2*	0.9%	2%
*KIT*	2%	1%
*FGF1*	0%	1%
*EGFR*	2%	0.4%
*BRAF*	0.6%	5%
*PTEN*	0.6%	6% ^d^
*RB1*	2%	7% ^d^
*NF1*	4%	6% ^d^
*ETV4*	0%	0.5%
*FOXM1*	0%	5%
*LSR*	0%	8%
*CD9*	0.3%	6%
*RAB11FIP4*	0%	3% ^d^
*FGFRL1*	0%	3%

^a^ The Cancer Genome Atlas Research Network [16]; ^b^ Other genes with copy number alterations exceeding a frequency of 15% include *NDRG1*, *EPPK1*, *PLEC*, *RECQL4*, *PTK2*, *EXT1*, and *RAD21*; ^c^ Promoter hypermethylation is also present in 12% of *BRCA1*; and ^d^ Represented by all or mostly all copy number deletions.
